# Composition of nanoclay supported silver nanoparticles in furtherance of mitigating cytotoxicity and genotoxicity

**DOI:** 10.1371/journal.pone.0247531

**Published:** 2021-02-25

**Authors:** Chih-Hao Chang, Yu-Hsuan Lee, Zhen-Hao Liao, Mark Hung-Chih Chen, Fu-Chuo Peng, Jiang-Jen Lin

**Affiliations:** 1 Department of Orthopedics Surgery, National Taiwan University Hospital and National Taiwan University College of Medicine, Taipei, Taiwan; 2 Institute of Polymer Science and Engineering, National Taiwan University, Taipei, Taiwan; 3 Institute of Toxicology, National Taiwan University College of Medicine, Taipei, Taiwan; Addis Ababa Science and Technology University, ETHIOPIA

## Abstract

Silver nanoparticle (Ag-NP) is well known for its high antibacterial efficacy. However, its toxicity toward mammalian cells is still a concern in clinical applications. The aim of our study was to evaluate the composition effects of Ag-NP supported by silicate nanoplatelet (NSP) with respect to the cytotoxicity and genotoxicity, and was in reference to the poly (styrene-co-maleic anhydride)-supported Ag-NP (Ag-NP/SMA). The NSP at the geometric dimension of averaged 80 x 80 x 1 nm^3^ was prepared from the exfoliation of natural clays and used to support different weight ratio of Ag-NP. The supporting limitation of NSP on Ag-NP was below the weight ratio of 15/85 (Ag-NP to NSP), and the detached Ag-NP from the Ag-NP/NSP (30/70) and Ag-NP/SMA hybrids were observed by TEM. Ames test was performed to assess the mutagenic potential of different compositions of Ag-NP/NSP, only Ag-NP/NSP (30/70) and Ag-NP/SMA hybrids exhibited mutagenicity when the concentration was 1.09 ppm or higher. In viewing of cytotoxicity using MTT tests toward HaCaT cells, the IC30 of Ag-NP/NSP (1/99, 7/93 and 15/85) were 1416.7, 243.6, and 148.9 ppm respectively, while Ag-NP/SMA was 64.8 ppm. The IC30 of Ag-NP/NSP (1/99, 7/93 and 15/85) were at least 833, 78 and 7 folds higher than their corresponding minimum inhibitory concentrations (MIC) respectively, and whereas Ag-NP/SMA was 6.4 folds. The Ag-NP/NSP and Ag-NP/SMA hybrids had been further investigated for genotoxicity by chromosomal aberrations and *in vivo* micronucleus assay within the concentration at IC10 and IC30, only Ag-NP/SMA showed a higher frequency of chromosomal aberrations. Our findings indicated that the viability of utilizing the NSP to maintain Ag-NP for antimicrobial activity, and the high-surface area of NSP served as an excellent support for associating Ag-NP and consequently rendering the mitigation of the inherent toxicity of Ag-NP in clinical uses.

## Introduction

Nanomaterials with different physical properties and compositions [1–3] may give rise to new applications in biomedicines [4], drug-delivery agents [5, 6], or biosensors [7–9]. Silver nanoparticles (Ag-NP) have been identified as effective antimicrobial agents and have been widely utilized as bactericides [10, 11] in many medical fields such as wound dressings, catheters, implants coating, and bone cements [12–14]. The antimicrobial mechanisms of Ag-NP involve the process of adhesion onto the cell surface (impairment of membrane structure and permeability), penetration of the cell and dysfunction of intracellular structures (mitochondria and ribosomes), induction of reactive oxygen species (ROS) and denaturation of biomolecules (protein, lipid and DNA), and modulation of signal transduction pathways [15–20]. Although Ag-NP are less reactive than silver ions for clinical and therapeutic applications [21], there is still a serious concern on the toxicity toward mammalian cells and the environment [22–26]. Toxic effects to the human body are caused by absorption through skin, lung, or digestive systems [23, 24, 27], and *in vitro* studies have also indicated that Ag-NP might enter mammalian cells and elicit cytotoxicity and genotoxicity [28, 29]. For clinical applications of Ag-NP, an important issue is to determine the balance between bactericidal efficacy and minimum harm to mammalian cells.

The physicochemical characteristics of Ag-NP, such as particle sizes, shapes, dispersed states and stability are the important parameters for achieving high antimicrobial performance. The Ag-NP in suspension tend to aggregate into clusters or precipitation due to ionic and van der Waals attraction among the particles; thus, they could possibly affect their biological responses, including antibacterial properties [16]. The methods for avoiding physical aggregation of Ag-NP or chemical degradation generally involve the added organic constituents as stabilizers such as polyvinylpyrrolidone (PVP) [30], polyvinyl alcohol (PVA) and bovine serum protein [31], polyethylene glycol (PEG) [32], gelatine [33], starch [34], and styrene maleic anhydride polymer (SMA) [35]. However, polymeric agent wrappings on Ag-NP could adversely reduce the surface activity of Ag-NP and the antibacterial activities [22, 26]. Therefore, the use of inorganic nanomaterials for supporting with Ag-NP can be a new approach for avoiding the uses of organic compounds to reduce side effects [36].

The silicate nanomaterials have the characteristics of low toxicity and high biocompatibility and uses in drug delivery [37, 38]. Their properties of water dispersion and high surface area render the clay suitable for interacting with inorganic nanoparticles. Among these silicate minerals, sodium montmorillonite (MMT) is composed of a multi-layered structure with the existence of metal counter ions such as sodium ion. Its cationic exchange capacity (CEC) is *ca*. 120 mequiv/100 g, which allowed ionic exchange reaction and organic intercalation of the layered structures [39]. The exfoliation process of multi-layered sodium MMT allowed to produce randomized nanoscale silicate platelets (NSP) with the physical properties of high surface area at *ca*. 750 m^2^/g and 18,000 ionic charges per platelet unit [40–42]. The silicate platelets with the dimension of 80 x 80 x 1 nm^3^ have high-surface affinity for interacting with cell membrane and inhibiting bacterial growth [43, 44]. Moreover, NSP has demonstrated its safety in cytotoxicity and genotoxicity due to its biocompatibility with mammalian cells [45, 46]. These advantages of naturally occurring NSP with high surface area for interacting with Ag-NP may be the ideal support material for clinical uses. Previously, we have reported the uses of NSP for supporting Ag-NP as the conjugated hybrids for the applications, including the treatment of silver-resistant bacteria [47], infected wound healing [45] and *Salmonella* infection [48]. In the previous work, it was defined the hybrids of various Ag-NP/NSP composition ratios and their difference in enhancing antimicrobial activity [49]. However, the proper compositions of Ag-NP/NSP by their different weight ratios during the synthesis of the hybrids have not been determined with respect to the balance between antimicrobial efficacy and biosafety. Hence, the purpose of this study is to thoroughly investigate the safety issues with respect to cytotoxicity and genotoxicity *in vitro* and *in vivo*. The polymer-supported Ag-NP/SMA was the reference for the meaningful comparison of the relative toxicity and antibacterial efficacy.

## Materials and methods

### Preparation of Ag-NP/NSP and Ag-NP/SMA

The NSP with an average dimension of 80 × 80 × 1 nm^3^ in polygonal shape was prepared by exfoliating the multi-layered structure of sodium MMT, according to the procedures described previously [40, 42, 50]. The pristine MMT was supplied by Nanocor Co. (USA). Using the exfoliated NSP as the supports, the Ag-NP/NSP with the composition weight ratios of 1/99, 7/93, 15/85, and 30/70 were prepared. The procedures for the preparation of these nanohybrids and their characterization have been described previously [49]. The Ag-NP dispersed in the SMA copolymer were prepared at the weight ratio of 7/93 in this study. According to the procedures reported previously [35], the SMA (0.93 g) was mixed with an aqueous solution of silver nitrate (AgNO_3_, 0.11 g) (Sigma-Aldrich, USA) dissolved in deionized water (52.0 g), followed by the addition of sodium borohydride (NaBH_4_, 0.03 g) (Sigma-Aldrich, USA) under a nitrogen atmosphere. The UV spectrometer (Hitachi U-4100, Japan) was utilized for measuring the maximum absorbance at 400 nm for Ag-NP/NSP and 410 nm for Ag-NP/SMA. The transmission electron microscope (TEM, JOEL JEM-1230, Japan) was employed to measure particle sizes and the size distribution was estimated from 100 particles.

### Silver particle detachment of Ag-NP/NSP by using TEM

*Escherichia coli (E*. *coli)* strains DH5α were incubated in Luria-Bertani (LB) broth (Gibco, USA) at 37 °C. After the cells were grown to the log phase (OD600 of 0.3 to 0.5). Ag-NP/NSP or Ag-NP/SMA was added to a 10 mL aliquot of the solution at approximate 1 × 10^7^ CFU/mL (colony formation unit/mL) at the final solution of 5 ppm and allowed standing for bacterial growth for 2h. The bacteria were collected by centrifugation (10,000 rpm, 5 min, 4 °C) and then fixed in 2.5% glutaraldehyde (Sigma-Aldrich, USA) solution for 4h. Dehydration of the cells was carried out by exposing the sample for 10 minutes to a series of 50, 60, 80, 90 and 100% ethanol/PBS solutions. The bacteria were finally embedded in LR White^™^ resin (Sigma-Aldrich, USA) and allowed to polymerize in an oven at 60 °C for 24h. The samples were cut into slices and analysed the interior of the bacteria by using TEM (JOEL JEM-1230, Japan).

### Ames test

Ag-NP/NSP and Ag-NP/SMA were evaluated for mutagenicity by the Ames mutagenicity assay using the plate incorporation method [51]. Histidine-required *Salmonella typhimurium* strains, TA98, TA100, TA102 and TA1535 were tested either with or without S9 (metabolic activation fraction mixture to mimic the mammalian metabolic conditions). The test strains were prepared as a homogeneous suspended solution of Ag-NP/NSP or Ag-NP/SMA with the concentration of Ag-NP at 0.55, 1.09, 2.19, 4.38 and 8.75 ppm with the S9 or plain buffer in the final volume of 0.6 mL. The doses were selected not to inhibit the growth of *S*. *typhimurium* strains. After incubation at 37 °C for 1h, 2 mL of molten top agar was added to cover plating aliquot (0.1mL) and spread over on a minimum histidine agar plate. His^+^ revertant colonies were counted in triplicate after incubation for 48h. Negative control (NC) was tested with double-distilled water (ddH_2_O). Positive control (PC) mutagen incubation without S9 treatment was sodium azide (8 μg/plate) (Sigma-Aldrich, USA) for TA1535 and mitomycin C (1 μg/plate) (Sigma-Aldrich, USA) for TA98, TA100 and TA102; with S9 treatment was 2-aminoanthracene (4 μg/plate) (Sigma-Aldrich, USA) for TA1535 and benzo[a]pyrene (1 μg/plate) (Sigma-Aldrich, USA) for TA98, TA100 and TA102.

### Cytotoxicity assay of Ag-NP/NSP and Ag-NP/SMA

Human keratinocyte cell line (HaCaT) was cultured in Dulbecco’s Modified Eagle Medium (DMEM, Gibco, USA) supplemented with 2 mM L-glutamine (Sigma-Aldrich, USA) at pH 7.3, 10% (v/v) FBS (Gibco, USA) and 1% (v/v) penicillin/streptomycin/amphotericin (Sigma-Aldrich, USA) at 37 °C under 5% CO_2_. 2 × 10^5^ cells were incubated in l mL culture medium for 24h before the treatment. The tests for Ag-NP/NSP and Ag-NP/SMA were set with the sublethal Ag-NP concentration of 1, 5, 10, 20 and 40 ppm, the concentration of control experiments of NSP and SMA copolymer were based on their corresponding concentration in Ag-NP/NSP and Ag-NP/SMA treated groups. After 3h and 24h treatments, 3-(4, 5-dimethylthiazol-2-yl)-2, 5-diphenyl tetrazolium bromide (MTT, Sigma-Aldrich, USA) solution was added to each well (100μg/well) and incubated at 37 °C for 2h. The medium was removed, and the dimethyl sulfoxide (DMSO, Sigma-Aldrich, USA) was added to dissolve the formazan crystals. Then, the purple solution from each well was transferred to a 96-well. The spectrophotometric absorbance at 570 nm was measured by a multi-well ELISA reader (DV-990-BV, Italy) for evaluating cell viability.

### Chromosome aberration test *in vitro*

The procedure of chromosome aberration test was adopted as the protocol from M. Ishidate Jr. [52]. Chinese hamster ovary cells (CHO) were cultured for density of 4 × 10^5^ cells/plate in 6 cm dish in 2 mL DMEM (Sigma-Aldrich, USA) for 24h. Based on the MTT tests, the dosages result in 90, 80 and 70% cell viabilities were performed in the chromosome aberration assay. Treatment of Ag-NP/NSP (1/99, 7/93 and 15/85) and Ag-NP/SMA were depended on their concentration of IC10 and IC30 (Table 3) for 20h, respectively. The NC group was tested with ddH_2_O; and the PC group without S9 was treated with mitomycin C (0.07 μg/mL) (Sigma-Aldrich, USA), and the PC group with S9 was treated with casein phosphopeptides (10 μg/mL) (Sigma-Aldrich, USA). The treated CHO cells were then fixated on the glass slides by (10 μg/mL) (Sigma-Aldrich, USA) colchicine and stained by Giemsa solution (5%) (Sigma-Aldrich, USA) for 1h. Cells in metaphase were screened for chromosomal aberrations, including chromatid (isochromatid) breaks, chromatid (isochromatid) gaps and dicentric, ring, lagging chromosomes.

### Micronucleus assay *in vivo*

All animal experiments were conducted in accordance with policies of the Institutional Animal Care and Use Committee (IACUC) of National Taiwan University College of Medicine (NTUCM). The protocols used in this study were approved by IACUC (approved no. 20130364). ICR male and female mice (8 weeks old and 25 g weight) were acquired from Laboratory Animal Center (LAC), NTUCM. All mice were acclimatized for 1 week before being fed the solutions of Ag-NP nanocomposites at the dose of 5000 mg/kg body weight. In NC group, the mice were fed pure water while the PC group mice were injected intraperitoneally (i.p.) with mitomycin C (Sigma-Aldrich, USA) at dose of 1 mg/kg. Peripheral-blood cells were collected over the periods of 24h, 48h, and 72h. Micronucleus formations in the polychromatic erythrocytes were counted under microscope by Giemsa staining (Giemsa Stain Kit, Abcam, USA).

### Statistical analysis

Each experiment was repeated in triplicate and data was shown as mean±SD (standard deviation). Statistical differences are evaluated by Student’s t-test. Differences are regarded as significant at p < 0.05.

## Results

### Mutagenicity of Ag-NP/NSP and Ag-NP/SMA

**Fig 1:**
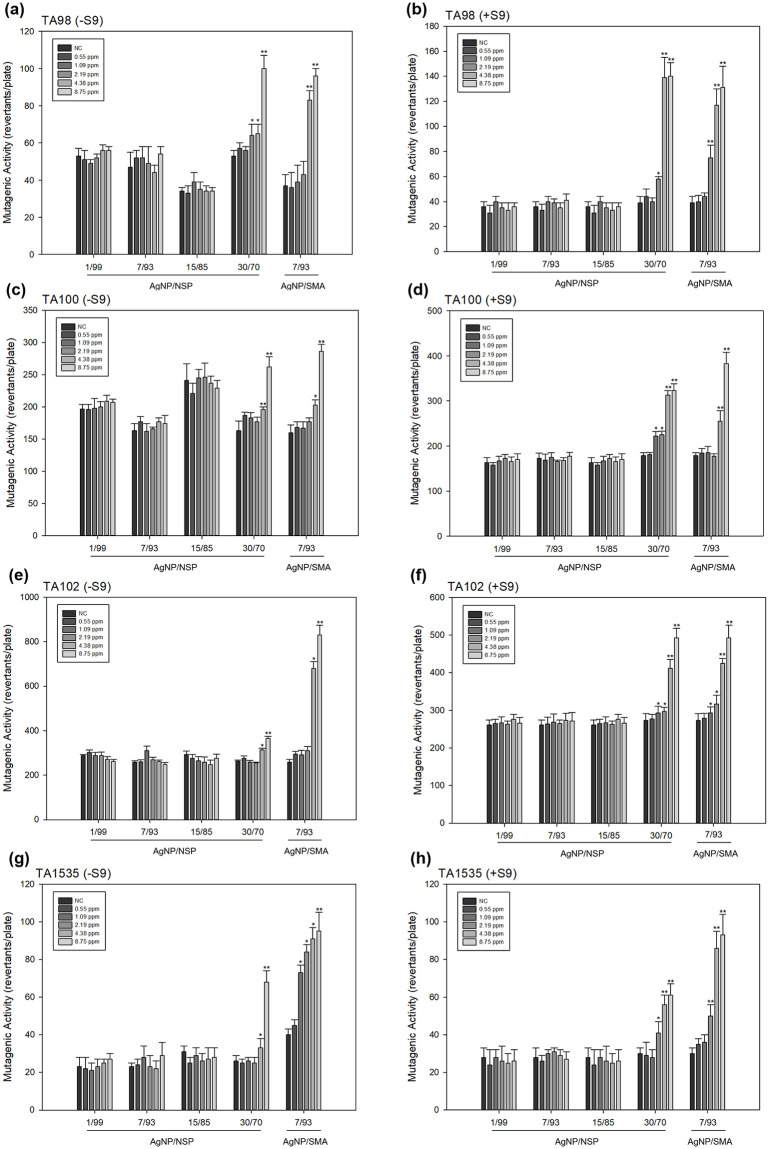
Mutagenic activity of Ag-NP/NSP and Ag-NP/SMA on *Salmonella typhimurium* (TA98, TA100, TA102 and TA1535) by Ames test with and without S9. (a), (c), (e) and (g) were the results of Ames test without S9 (-S9) in TA98, TA100, TA102 and TA1535 strains, respectively; (b), (d), (f) and (h) were the results of Ames test with S9 (+S9) in TA98, TA100, TA102 and TA1535 strains, respectively. Each bar was the mean with standard deviation for three independent experiments. NC meant the negative control by using distilled water only. * denoted P < 0.05 as obtained by Student’s t test, where the statistical significance between the treated groups and negative control were analysed for each concentration. ** denoted P < 0.01 as obtained by Student’s t test, where the highly statistical significance between the tested groups and negative control were analysed for each concentration.

As shown in Fig 1(a) to 1(h), the mutagenicity was investigated, including the results of Ames test with (+S9) or without S9 (-S9) in TA98, TA100, TA102 and TA1535 strains. A similar trend was found while Ag-NP/NSP of 30/70 and Ag-NP/SMA significantly increased in revertant colonies at the dose over 1.09 or 2.19 ppm/plate, whereas Ag-NP/NSP of 1/99, 7/93 and 15/85 showed no mutagenic responses either with or without S9. Ames test provides the initial screening on the selecting non-mutagenic compositions of Ag-NP/NSP at 1/99, 7/93 and 15/85 for further genotoxicity tests in eukaryotic systems.

### Silver particle detachment of Ag-NP/NSP by TEM observation

**Fig 2:**
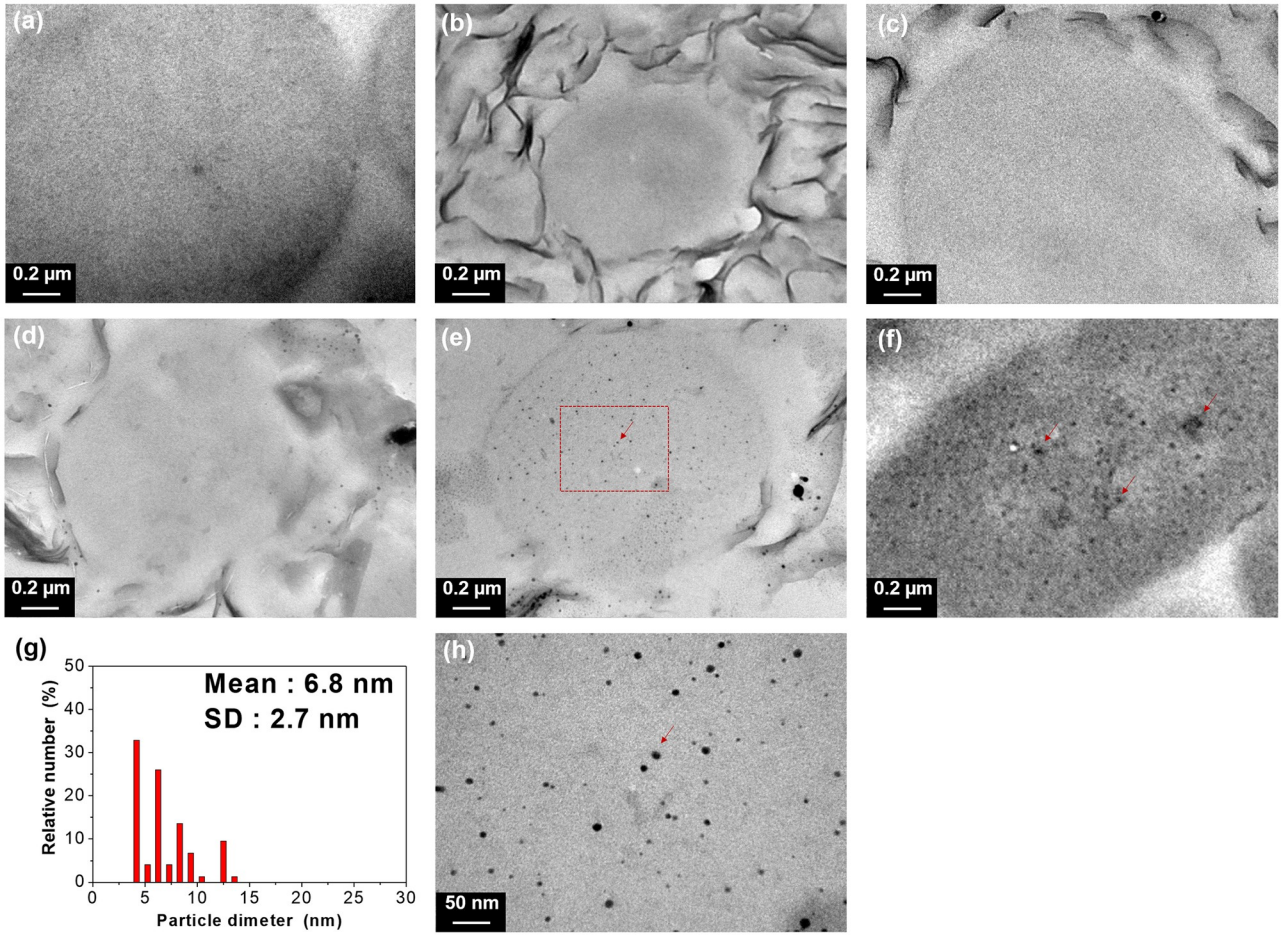
Transmission electron micrographs of the *E*. *coli* morphology after incubation with Ag-NP/NSP and Ag-NP/SMA hybrids. (a) No treatment, (b) Ag-NP/NSP (1/99), (c) Ag-NP/NSP (7/93) and (d) Ag-NP/NSP (15/85). Representative Ag-NP were labelled as red arrows in (e) Ag-NP/NSP (30/70), and (f) Ag-NP/SMA (7/93). Enlarged view of the area in (e) is shown in (h) with statistic size distribution of Ag-NP (g).

The close-up TEM micrographs in Fig 2 exhibited the direct observation of the interaction between Ag-NP and *E*. *coli* cells. The average length of *E*. *coli* cells at 1–3 μm and the morphology of cell walls were visualized. After the treatments of Ag-NP/NSP with different compositions of 1/99, 7/93 and 15/85, none of the Ag-NP entering inside of the *E*. *coli* cells was observed (Fig 2b–2d), and the results were consistent with the cells without any treatment (Fig 2a), which implied that there was neither leaching Ag-NP from NSP surface nor Ag-NP penetrating into the cell body. In contrast, Ag-NP appeared inside of *E*. *coli* cell body in the treatments of Ag-NP/NSP (30/70) and Ag-NP/SMA (Fig 2e and 2f), indicating the Ag-NP penetration into cells (the arrow points). In order to further compare to the originally prepared size was shown in average 17 ± 2.9 nm (as shown in Table 4), the Ag-NP found inside the bacteria cells were those of the smaller ones at the mean size of 6.8 ± 2.7 nm (Fig 2g and 2h) in the case of Ag-NP/NSP (30/70). It appears that the Ag-NP entering into bacterial cells were those from the detachment of the NSP supports. In the case of Ag-NP/SMA, the SMA wrapping failed to prevent the Ag-NP from entering into cells (Fig 2f).

### Cytotoxicity of Ag-NP/NSP and Ag-NP/SMA

**Fig 3:**
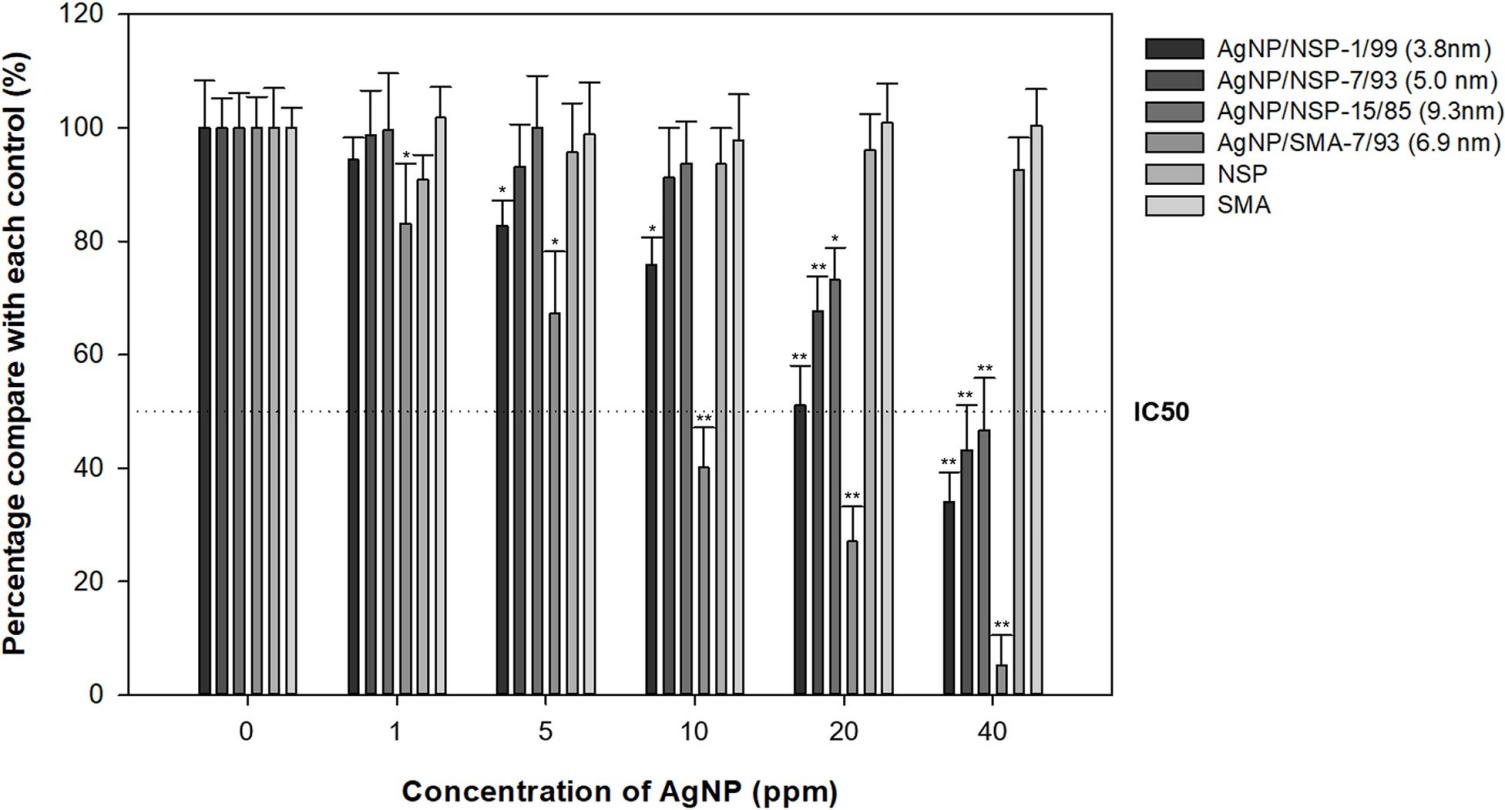
Cytotoxicity of Ag-NP/NSP at different weight compositions and Ag-NP/SMA using the MTT assay. HaCaT cells were incubated with 0–40 ppm Ag-NP concentration of nanocomposites for 24h. The percentage of survival cells was relative to the negative control cells (0 ppm) and by the mean ± standard deviation of four independent experiments. *denoted P < 0.05 as obtained by Student’s t test, where the statistical significance between the treated groups and control group (0 ppm) were analysed for each concentration, respectively. ** denoted P < 0.01 as obtained by Student’s t test, where the highly statistical significance between the tested groups and control group (0 ppm) were analysed for each concentration, respectively.

The cytotoxicity of Ag-NP/NSP and Ag-NP/SMA was examined under the exposed time of 24h toward HaCaT cells as shown in Fig 3. Three compositions of Ag-NP/NSP (1/99, 7/93 and 15/85) were tested in comparison with Ag-NP/SMA (7/93). The results of MTT assay indicated that Ag-NP/NSP of the three compositions all exhibited higher cell viability than Ag-NP/SMA in different Ag-NP concentrations. Based on the control experiments, both NSP and SMA themselves were not contributed to the cytotoxic effects. Moreover, the calculated sublethal concentration of 30% (IC30) values of Ag-NP/NSP (1/99, 7/93 and 15/85) were 1416.7, 243.6, and 148.9 ppm, respectively, whereas the IC30 value of Ag-NP/SMA was 64.8 ppm (Table 1). The IC30 of Ag-NP/NSP (1/99, 7/93 and 15/85) were at least 833, 78, and 7 folds higher than their corresponding minimum inhibitory concentrations (MIC) respectively, and whereas Ag-NP/SMA was 6.4 folds (as shown in Table 4). Based on the results of cytotoxicity, the use of inorganic NSP as support showed the mitigation of the Ag-NP toxicity toward HaCaT cells in contrast to the organic SMA.

**Table 1:** The IC10 and IC30 of Ag-NP/NSP and Ag-NP/SMA.

	Cell viability (%)	Ag-NP/NSP (Ag-NP) (ppm)			Ag-NP/SMA(Ag-NP) (ppm)
		1/99	7/93	15/85	7/93
IC10	90	172.7 (1.7)	125.1 (8.8)	70.7 (10.6)	9.5 (0.7)
IC30	70	1416.7 (14.2)	243.6 (17.0)	148.9 (22.2)	64.8 (4.5)

### Genotoxicity of Ag-NP/NSP and Ag-NP/SMA

The chromosome aberration assays were conducted to compare the genotoxicity of Ag-NP/NSP with one of Ag-NP/SMA. As summarized in Table 2, various types of chromosomal aberrations such as gaps and breaks of chromatid were observed in all Ag-NP/SMA, showing significantly higher frequency of chromosomal aberrations than the negative control. By comparison, all of Ag-NP/NSP tests were similar to the negative control. The micronucleus assays were examined for producing micronuclei during cell division with respect to the chromosomal breakage and mitotic spindle damage (Table 3). The frequencies of micronucleated polychromatic erythrocytes (MNPCEs) were evaluated after feeding with the hybrids for 36h, 48h, and 72h. The treatments of Ag-NP/NSP in three compositions as well as Ag-NP/SMA showed no increase in the frequency of micronuclei. No influence on genotoxicity *in vivo* for both groups of Ag-NP feeding was found.

**Table 2:** Genotoxicity of chinese hamster ovary (CHO) cell on chromosome aberration after treating Ag-NP/NSP and Ag-NP/SMA.

Treatment	w/w	Dosage	Aberration/100 cells	
			0	0
Negative control^a^			2	3
Positive control^b^			11*	14*
Ag-NP/NSP	1/99	IC10	1	3
		IC30	1	3
	7/93	IC10	1	4
		IC30	1	2
	15/85	IC10	2	2
		IC30	2	3
Ag-NP/SMA	7/93	IC10	5*	7*
		IC30	10*	13*

Negative control: ddH_2_O.

Positive control: *mitomycin C* (0.07μg/mL).

*Significant difference from the negative control (Student’s t-tests; P<0.05).

**Table 3:** *In vivo* genotoxicity of micronucleated polychromatic erythrocytes (MNPCEs) in mice after treating Ag-NP/NSP and Ag-NP/SMA.

Dose (5000 mg/kgw)	w/w	Frequencies of MNPCEs^a^		
		36h	48h	72h
Negative control		2.9±0.89^c^	3.2±0.83	3.4±0.67
Positive control^b^		9±0.71*	15.8±2.77*	17.3±2.49*
Ag-NP/NSP	1/99	2.4±0.99	3.5±0.97	3.8±0.63
	7/93	2.1±0.31	3.4±0.83	3.6±1.35
	15/85	2.8±0.94	3.3±1.10	3.4±0.40
Ag-NP/SMA	7/93	3.1±0.94	3.1±1.04	3.3±1.12

^a^ Two-thousand polychromatic erythrocytes were analyzed per animal, for a total of 20,000 cells per group.

^b^ Positive control group received i.p. injection of l mg/kg of mitomycin C.

^c^ Date collected by mean ± standard deviation for three independent experiments.

*Significantly different from the negative control (Student’s t-tests; P<0.05).

## Discussion

For medical devices, although Ag-NP have many benefits as the most widely used antimicrobial nanomaterials and the better ones for inhibiting the undesirable growth of bacterial biofilms, but their overall toxicity remains unknown. In this study, the composition effects of Ag-NP supported by silicate nanoplatelet (NSP) with respect to the cytotoxicity and genotoxicity were evaluated and were in reference to the SMA-supported Ag-NP.

We investigated the mutagenicity by Ames tests performed using four *Salmonella* strains. The results indicated the negative mutagenicity from the Ames tests of Ag-NP/NSP (1/99, 7/93 and 15/85) in all strains; and, in contrast, Ag-NP/NSP (30/70) was observed a mutagenicity. Studies showed that Ag-NP which enter the cells through diffusion or endocytosis can cause mitochondrial dysfunction and generation of ROS. Furthermore, they can damage proteins and nucleic acids, inhibiting cell proliferation. The feature of Ag-NP size is associated with the ability of NPs for free diffusion through nuclear pore complexes [53]. At the G2/M phase, interaction of nanosilver with DNA also leads to cell cycle arrest. With the intensive affinity for van del Waals force association with Ag-NP, the involved NSP with high surface area and SiO^-^Na^+^ ionic charges further impede the tendency of penetrating into cell nucleus. The series of Ag-NP/NSP with various weight-ratios elucidated the NSP supporting capacity. Beyond the loading capacity of NSP in the cases of 30/70 of Ag-NP/NSP, the detached Ag-NP from NSP surface became the cause of raising mutagenicity. Further evidence was revealed by the TEM images after treating *E*. *coli* cells with Ag-NP/NSP, visualizing the detached Ag-NP particles inside the cell body.

The compositions of Ag-NP/NSP (1/99, 7/93 and 15/85) with negative mutagenicity were further investigated. In Table 2, there were no significant chromosomal aberrations at 1.5 to 72 ppm for 20h treatments in the chromosome aberration test on CHO cells of three Ag-NP/NSP. Previous SEM images were shown that NSP tended to accumulate and adhere on the CHO cell surface due to its high surface affinity and thin platelet shape [46]. Both interactions of tightly attaching Ag-NP on NSP and the NSP adhering with cell surface prevented the Ag-NP entrance into cell nucleus and causing DNA damage. In the case of Ag-NP/SMA (7/93), none of strong supporting effect as NSP and Ag-NP freely entering cells and causing a positive genotoxicity as indicated in chromosome aberration test was found in the SMA polymers (Table 2). For SMA wrapped Ag-NP, the particle entrance inside the cell body and the correlation of positive genotoxicity were identified. Therefore, the genotoxicity and the NSP supporting effect are correlated with each other, which is caused by the entrance of free Ag-NP into the nucleus of mammalian cells.

Although the difference in genotoxicity between Ag-NP/NSP and Ag-NP/SMA has been demonstrated *in vitro* tests, the feeding tests did not show a significant difference by the evaluation of micronucleated polychromatic erythrocytes (MNPCEs) (Table 3). All tests of Ag hybrids showed a similar value of MNPCE frequencies to the negative control. The similar results were reported previously, there were no significant differences in the MNPCEs when the rat oral toxicity of Ag-NP (particle size: 60 nm) over a period of 28 days at 1000 mg/kg weight/day [54]. All examined mice were found none of significant abnormality with respect to their appearances and body weights. The data indicated that oral administrating Ag-NP/NSP and Ag-NP/SMA at high dose of 5000 mg/kg weight did not elicit acute toxicity and hence no genotoxicity *in vivo*.

Several studies have reported that the Ag-NP size is a main cause of the toxicity of nanoparticles, and especially the particle size below 10 nm is more toxic towards bacteria [55]. Ag-NP can interact with biomolecules such as protein, lipid, and DNA by penetrating into the cytoplasm. In some cases, Ag-NP can generate reactive oxygen species (ROS) such as hydrogen peroxide (H_2_O_2_), hydroxyl (OH^–^) and superoxide (O2^–^) radicals that induce oxidative stress and damage to proteins and nucleic acids while interacting with the respiratory enzyme system [56]. Ag-NP/NSP hybrids (1/99, 7/93 and 15/85) and Ag-NP/SMA (7/93) were probed with their cytotoxicity on HaCaT cells (Fig 2). Ag-NP/SMA (7/93) had exhibited a higher toxicity than that of Ag-NP/NSP hybrids (1/99, 7/93 and 15/85). The results indicated that the NSP support is superior to an organic SMA used as the stabilizer when interacting with Ag-NP in mitigating the Ag-NP cytotoxicity. While the antimicrobial activities of the series of Ag-NP/NSP were reported [49], the cytotoxicity and genotoxicity are summarized in Table 4. It appeared that all of Ag-NP/NSP at the compositions of 1/99 and 7/93 exhibited none of genotoxicity and high cell viabilities at MICs. In particular, Ag-NP/NSP (1/99) exhibited 99% cell viability at MIC (3.9 ppm). Although Ag-NP/NSP (30/70) showed a decent antimicrobial efficacy (MIC = 14 ppm) with Ag-NP/NSP (15/85) (MIC = 19 ppm), the detached Ag-NP in the samples of Ag-NP/NSP (30/70) may enter into the *E*. *coli* cells and caused genotoxicity. Complex factors, such as cell types, cell culture media and Ag ions release, might affect the Ag-NP/NSP-induced cytotoxicity [16, 56, 57]. Further research into verifying the cellular ROS levels in each cell line upon different sizes of Ag-NP/NSP is required. Generally speaking, the generation of ROS may determine whether Ag-NP-induced cytotoxicity is produced.

**Table 4:** Summary of antimicrobial efficacy, cell viability, and genotoxicity of Ag-NP/NSP and Ag-NP/SMA.

Ag-NP hybrids (w/w)	Ag-NP size (nm)^a^	MIC (ppm, 24 h)^b^	IC30 (ppm, 24 h)^c^	IC30 /MIC	*In vitro* genotoxicity
Ag-NP/NSP					
1/99	3.8±1.8^d^	1.7^d^	1416.7	833.4	negative
7/93	4.5±1.5^d^	3.1^d^	243.6	78.6	negative
15/85	9.3±2.3^d^	19.0^d^	148.9	7.8	negative
30/70	17±2.9^d^	14.4^d^	N.D.	N.D.	positive
Ag-NP/SMA					
7/93	6.9±2.1	23.1	64.8	6.4	positive

^a^ Based on TEM images.

^b^ MIC: minimum inhibitory concentration for *E*. *coli*.

^c^ IC30: 30% of maximal inhibitory concentration for HaCaT cells.

^d^ Data was cited from references (Wei, Yen et al. 2013).

The analyses of Ag-NP particle size was summarized in Table 4. Interestingly, the smaller Ag-NP rendered a higher antibacterial activity in the hybrids at the compositions below 15/85. Since there were no Ag-NP entering inside *E*. *coli* cell bodies from the examination of TEM images (Fig 2), the higher antibacterial effect of Ag-NP might not have better penetration ability into *E*. *coli*. because of the smaller particle size. Membrane damage, resulting in the leakage of cellular contents and eventual cell death was done due to direct contact of Ag-NP with large surface areas on a bacterial cell wall [16, 57–59]. Different sizes of Ag-NP tethered on NSP surface could interact with the cell membrane and resulted impairments in lipid bilayer leading to different level of ROS [20, 36, 47, 60].

In our previous studies, the uses of nontoxic SMA polymer for supporting Ag-NP did reduce the cytotoxicity, but still induced hemolysis [35]. Non-specific proteins might be bound with the surface of negatively charged Ag-NP/SMA, which promotes cellular uptake [61]. For Ag-NP/SMA (7/93) in this study (Table 2), the IC30 (64.8 ppm) was closer to its MIC (23.1 ppm) in the anti-*E*. *coli* tests. In addition, Ag-NP/SMA (7/93) caused DNA damage in terms of Ames tests and chromosomal aberration. These studies demonstrated that the inorganic NSP is superior to SMA for supporting Ag-NP owing to the high adhering ability of NSP towards both of Ag-NP and cell membrane to prevent the free Ag-NP entering the cell bodies. Based on our previous SEM results (Li, Wei et al. 2010), the CHO cells were found to have aggregation of NSP on cell surface after 24h incubation. The adhesion between the cell surface of anionic character and the sodium ion charged NSP is primarily stable, and consequently prevents the cellular uptake of NSP-supported Ag-NP. The assumption is confirmed by the observation of FITC-labelled NSP associated with the cells, by using the confocal images on human mesenchymal stem cell. The course and the outcome of NSP during the interaction with cells are shown in the images. In S1 Fig, the cell surface was first observed in the NSP self-aggregated clusters. After 3-day incubation, NSP was internalized into cytoplasm but not entering into nuclei (i.e., none of FITC signal observed within nucleus). The image indicated the entrance of Ag-NP into the cell nuclei was totally avoided by the presence of NSP. As a result, the genotoxicity was minimized owing to the presence of NSP in supporting Ag-NP.

**Fig 4:**
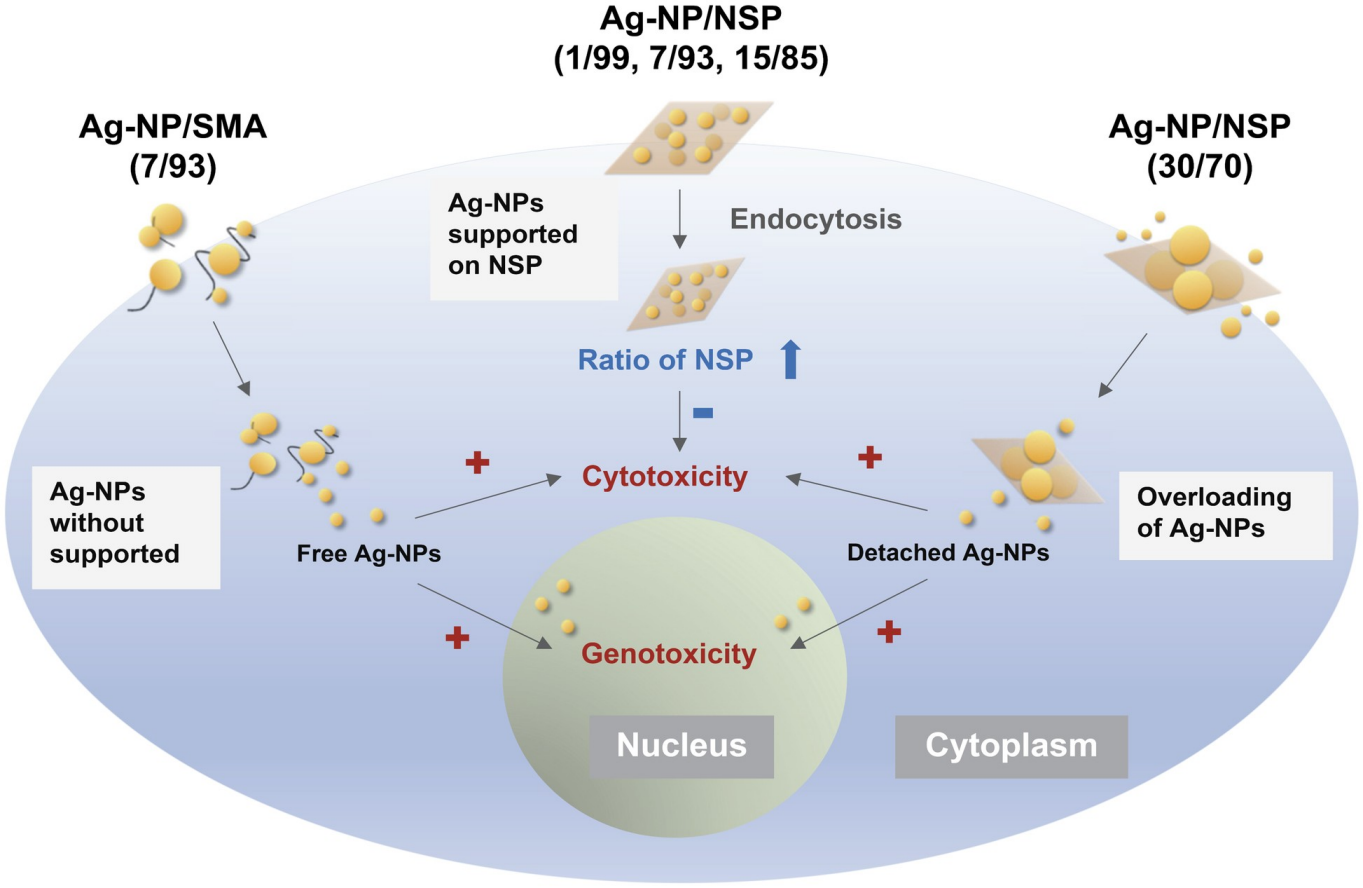
Conceptual illustration of the interactions between the hybrids, Ag-NP/SMA (7/93) or Ag-NP/NSP (ranging from 1/99–30/70) with cells.

In Fig 4, the comparison of interaction with cells between Ag-NP/SMA and Ag-NP/NSP is shown. The Ag-NP/NSP at the compositions ranging from 1/99 to 30/70 (w/w) were compared with Ag-NP/SMA (7/93, w/w) for the biosafety. The limitation of NSP supporting capability was found at the level of 15/85 (w/w). In other words, NSP could support Ag-NP up to 15% by weight. Beyond this weight limitation such as 30/70 (w/w), the Ag-NSP hybrids could not prevent the free Ag-NP penetrating into the cell body and causing genotoxicity. Hence, the superiority and the support effect of NSP for Ag-NP are established for mitigating the cell toxicity.

## Supporting information

S1 Fig Confocal image showing the interaction between FITC-tagged NSP and human mesenchymal stem cell. The mesenchymal stem cell was treated with NSP at 1 or 10 ppm in 1 to 6 days. The nuclei were stained with DAPI (blue) and the distributions of NSP was detected with FITC (green), and the observation was performed by laser scanning confocal microscopy (LSM780, Zeiss).
(TIF)
