# Supplementary figures and images for: Composition of nanoclay supported silver nanoparticles in furtherance of mitigating cytotoxicity and genotoxicity
Source: PLoS One. 2021 Feb 25;16(2):e0247531. doi: 10.1371/journal.pone.0247531 (PMC7906337; doi:10.1371/journal.pone.0247531)

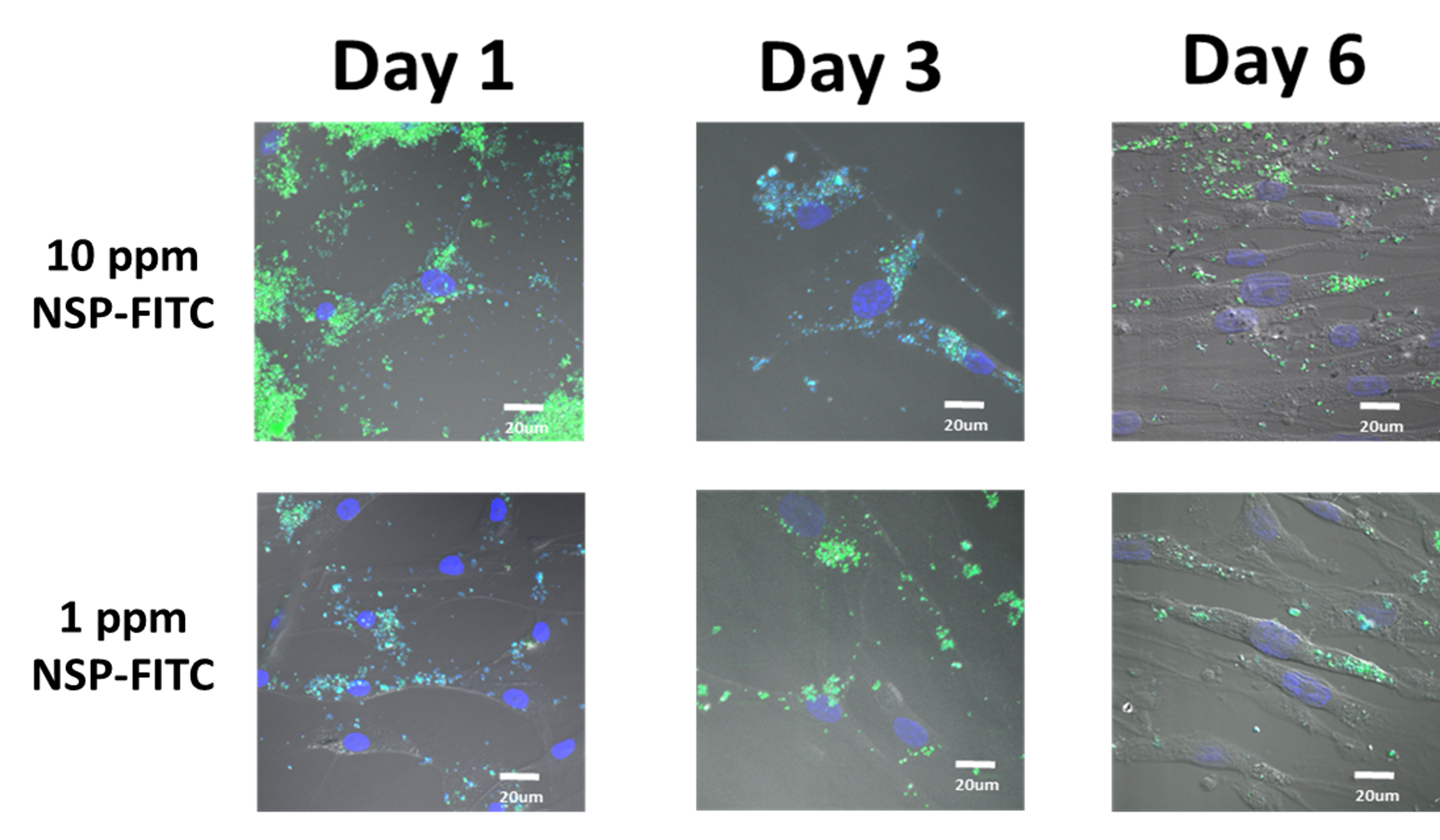

Supplement: S1 Fig — The mesenchymal stem cell was treated with NSP at 1 or 10 ppm in 1 to 6 days. The nuclei were stained with DAPI (blue) and the distributions of NSP was detected with FITC (green), and the observation was performed by laser scanning confocal microscopy (LSM780, Zeiss). (TIF) [file pone.0247531.s001.tif]
